# The Global Sweep of Pollution: Satellite Snapshots Capture Long-Distance Movement

**DOI:** 10.1289/ehp.116-a338

**Published:** 2008-08

**Authors:** Bob Weinhold

Towering smokestacks were a popular mid-twentieth-century “remedy” for industrial emissions. Pump the stuff high enough into the air, went the thinking, and the problem would go away. But evidence collected since then has strongly suggested that tall smokestacks are not sufficient to mitigate the effects of pollution—those pollutants eventually came down somewhere, dozens or thousands of miles away. In the November 2006 issue of *EHP*, for example, Morton Lippmann of the New York University School of Medicine and colleagues reported a strong link between nickel emitted from a very tall smokestack at a smelter in Sudbury, Canada, and acute heart rate changes in mice some 500 miles away. At the same time, we also now know that tall stacks are not necessary for pollutant emissions to waft great distances, as verified by scores of individual studies showing that one pollutant or another—such as ozone, particulate matter (PM), and sulfur dioxide (SO_2_)—can blow from country to country, and continent to continent.

Beginning in the 1980s and accelerating since about 2000, satellite surveillance with increasingly sophisticated instruments has enabled us to better visualize the complex fluctuations of several important pollutants as they ebb and flow around the planet. This new capability is partly serendipitous. “Most of these satellites weren’t designed to have an air quality focus,” says Terry Keating, an environmental scientist with the U.S. Environmental Protection Agency (EPA) and co-chair of the Task Force on Hemispheric Transport of Air Pollution, which was created in 2004 by the United Nations Economic Commission for Europe’s Convention on Long-Range Transboundary Air Pollution. “But we find ourselves with this stream of data, and we are figuring out how to use it,” he says.

One use has been a handful of pilot projects directly linking satellite-observed column-wise concentrations of atmospheric pollutants—that is, the concentration from the Earth’s surface to the top of the atmosphere—with concentrations at the ground level. “Only in the past ten years have we been able to advance epidemiological science with satellites,” says John Haynes, program manager for public health and aviation applications at the National Aeronautics and Space Administration (NASA). “This is truly a leap forward into the twenty-first-century science of epidemiology.”

## Launch of a New Field

Steady progress in the capabilities of satellites during the twentieth century provided glimpses of the planet from ever higher elevations. With the 1957 launch of the first satellite, Sputnik, by the Soviet Union, we gained our first view of the Earth from above the troposphere, the layer of the atmosphere that shimmies around the planet from the ground to elevations of about 4 to 12 miles.

The following year the United States created NASA. The study of atmospheric pollutants was far from the first priority in NASA’s early years; instead, the program focused on keeping the United States ahead of the Soviets in the Space Race. But the technological capabilities developed as part of the U.S. space exploration program have provided critical tools for beginning to observe the complex gyrations of various pollutants in the atmosphere.

Among the many other countries now involved in satellite programs targeting air pollutants are Brazil, Canada, China, India, Japan, and the 20 European countries that are the primary supporters of the European Space Agency (ESA). “More and more countries are getting aware of this problem all over the world,” says Claus Zehner, an ESA Earth observation applications engineer. “It is a growing theme in the satellite world.”

Tracking individual air pollutants can be quite a challenge. Satellite instruments use the unique spectral signature of a single chemical or class of chemicals to distinguish it from all the other substances floating around it. But scientists must also account for variables such as clouds, humidity, wind, and landforms; reflectance from land and ocean surfaces (which form the backdrop of what the instrument sees); time of day and season (which can affect formation of various compounds); and chemical reactions (which can alter the tracked compound before it reaches the ground, making it difficult to predict the exact chemical culprits of environmental health concern). In addition, there’s horizontal and vertical mixing of air masses at a wide range of temporal and spatial scales, including that caused when the upper troposphere mingles with the “planetary boundary layer,” where friction with the Earth’s surface alters airflow. And there’s one last complication—the mixing within the planetary boundary layer that determines the level of pollutants near the ground.

So far, scientists have begun to get the best handle on these variables for PM, nitrogen dioxide (NO_2_), carbon monoxide (CO), and formaldehyde. They’re also making some progress with ozone, but SO_2_ and most volatile organic compounds (VOCs) still pose a significant challenge. Other important pollutants that are known to be widely distributed—among them polycyclic aromatic hydrocarbons, ammonia, pesticides, and metals such as lead and mercury—are hard to distinguish from the PM to which they’re attached or have spectral signatures that are difficult to detect amidst all the complicating atmospheric conditions, says John Burrows, co-director of the Institute of Remote Sensing and Environmental Physics at the University of Bremen. Tracking such pollutants will require better instruments, Burrows says.

Indeed, we still have a long way to go before we can fully document and accurately forecast pollution streams and their effects on the ground. But data from satellites, aircraft, balloons, ground-level monitors, chemical transport models, and other tools are already improving our understanding of the global transport of pollutants as individual nations and international collaborations struggle to better address emissions and the effects they have on downwind countries and continents.

## A Fine View of Particulate Matter

PM is created by combustion of fuels in vehicles, power plants, domestic cooking, and industrial processes, and by natural processes such as wildfires, volcanic eruptions, wind-blown dust, and bursting of sea salt–laden bubbles at the ocean’s surface. PM can cause premature death and a range of respiratory and cardiovascular problems. Because of its various sources and residence time in the atmosphere, PM is highly variable in space and time. In contrast to gaseous pollutants, PM characterization must also include composition or type, particle size, and shape—all important dimensions for assessing detrimental effects of particulates on human health.

PM wasn’t considered a major long-range transport issue until about the mid-1990s. Even now, the 51 countries that participate in the United Nations Convention on Long-Range Transboundary Air Pollution are just beginning to address PM, discussing whether and how to add policies for this pollutant to the convention, says Andre Zuber, co-chair with Keating and a policy officer with the European Commission.

Satellite images indicate how far PM can travel. Almost all continents and regions can be a PM hotspot at one time or another. Some areas are a major source almost year-round, including central and southern Africa, eastern Asia, Indonesia, Europe, the eastern United States, and northern and central South America.

Satellites are also being used to quantify PM movement between continents. Hongbin Yu, an associate research scientist at the Goddard Earth Sciences and Technology Center, a joint research center of NASA and the University of Maryland Baltimore County, and his colleagues found that East Asian pollution sources added an average of about 15% to the non-dust PM burden already being generated in the United States and Canada from 2002 to 2005. These findings, published in the 22 April 2008 *Journal of Geophysical Research*, provide the first satellite-based estimate of transport of PM from East Asia to North America.

The study didn’t calculate the effects at ground level, which requires enhanced satellite capability to assess on a daily basis vertical structures of pollution plumes with high accuracy. “[That] process is still a bit fuzzy,” says Solar Smith, a research associate at the Center for Space Research at the University of Texas at Austin, who did not participate in the Yu study. “On a long-term scale, you can take the average over time and get a fairly reliable understanding of how [ground-level] air quality is changing. The challenge is individual scene analysis on individual days. We’re always working to improve algorithms.”

Using existing algorithms, an international team studied 26 locations in Sydney, Delhi, Hong Kong, New York City, and Switzerland, and found an overall 96% correlation between satellite measurements of atmospheric column PM loading and ground measurements of PM concentration, although there was significant variation caused by factors such as cloud cover, relative humidity, and circulation within the boundary layer. These results were published in the September 2006 issue of *Atmospheric Environment*. In the June 2008 issue of the same journal, Klaus Schäfer and colleagues reported a 90% correlation between satellite and ground measurements for fine particulates 2.5 μm in diameter or smaller (PM_2.5_) in the winter, but in the summer only a 48% correlation for PM smaller than 1 μm in diameter.

These variable results and those of other studies confirm that there is indeed still a bit of fuzziness in the processes of both measurement and calculation. The emerging data from lidar (the optical analog of radar) measurements taken aboard the CALIPSO (Cloud-Aerosol Lidar and Infrared Pathfinder Satellite Observation) satellite will help to get a clearer picture, says Yu. Satellite measurements with more extensive coverage than CALIPSO are needed in the future. But the results are becoming reliable enough—at least in some regions and seasons—that a handful of local projects are using or considering using PM data derived from satellites.

HELIX-Atlanta (Health and Environment Linked for Information Exchange in Atlanta, Georgia) is a five-county demonstration project whose principal investigator for its first half was Amanda Niskar, then with the Centers for Disease Control and Prevention (CDC). The project’s partners include NASA, the CDC, the EPA, the Georgia Environmental Protection Division, Kaiser Permanente of Georgia, the Georgia Institute of Technology, and Emory University. The goal is to improve knowledge of the link between PM_2.5_ and respiratory diseases among residents of the five-county area and improve forecasting of dangerous PM_2.5_ events as well as government, medical, and individual responses to them. Eventually this information could be extrapolated to other settings.

Satellites allow interpolation of PM_2.5_ data between EPA monitors on the ground. “That’s the great revolution, really,” Haynes says. “You get much better representation of PM_2.5_ concentrations over an area.” Another key benefit of the study is that the detailed individual-level health data provided by Kaiser Permanente (a health care provider) enables researchers to more accurately evaluate the role PM_2.5_ may be playing in complex medical conditions. HELIX researchers expect to soon publish their findings on the links between satellite and ground monitor data.

The related HELIX-Israel project is still in its organizational and fund-raising stages. Niskar, now a faculty member at the Tel Aviv University School of Public Health and director of HELIX-Israel, says the portion of the project that would use satellite data on air pollutants may begin as early as 2009. She says the Israeli project could be even more revealing than its Atlanta counterpart because the public health data available in Israel are of higher quality than those in the United States, and the network of ground monitors is denser, allowing for better cross-checking of satellite and ground data. If the project is successful, Niskar says she is eager to help other countries set up similar systems. She would also like to include other health outcomes beyond the initial respiratory and cardiovascular targets and other pollutants (such as ozone) once NASA and others develop accurate methods for determining ground-level concentrations.

Another project will look at links between PM_2.5_ and health outcomes such as stroke, blood pressure changes, cholesterol levels, deep vein thrombosis, and cognitive function. The REGARDS project (Reasons for Geographic and Racial Differences in Stroke), a multicenter study sponsored by the National Institute of Neurological Disorders and Stroke, already has 30,228 volunteers signed up in the lower 48 states and may be able to begin the satellite-related portion of its work as soon as 2009 if NASA funding comes through, says Leslie McClure, an assistant professor of biostatistics at The University of Alabama at Birmingham. “This is all very cutting-edge,” she says. “To be able to estimate exposure for someone in the plains of Kansas where monitors are scarce is an extraordinary power.”

## Closing In on Gases

Satellite tracking of several gaseous pollutants also is improving. One class of such pollutants is the nitrogen oxides (NO_x_), which are created primarily through vehicle, power plant, and industrial combustion processes, as well as by natural sources such as fires, soils, and lightning. NO_x_, despite their short lifespan in the lower atmosphere, play a key role in the formation of ground-level ozone, acid rain, and greenhouse gases. NO_2_, along with other toxic by-products of NO_x_ reactions, can cause health problems such as premature death, cancer, and respiratory and cardiovascular effects. NO_2_ is typically used by researchers and regulatory agencies as a surrogate for NO_x_ reaction by-products.

NO_2_ is one of the easier pollutants to track via satellite because of its spectral signature. “You can see it all the way down to the surface,” says Mark Schoeberl, project scientist for NASA’s Aura satellite, which carries several pollutant monitoring instruments. This has made it relatively simple to capture extensive satellite data about the global movement of this compound. There are a number of hotspots for the origin of NO_2_, and the pollutant can travel long distances. Portions of eastern China are major generators all year long, and moderately high emissions come from areas in Europe, the eastern United States, and central and southern Africa during much of the year.

In a study published in the 22 February 2008 *Journal of Geophysical Research*, Ronald van der A, a senior project scientist at the Royal Netherlands Meteorological Institute, and his colleagues reviewed satellite data from 1996 to 2006 for NO_2_ and found that some areas in China had seen an average annual increase of up to 29%. Portions of India, Iran, Russia, South Africa, and the central United States also had annual increases. But pollution reduction efforts may have contributed to small annual decreases in much of Europe and portions of the eastern United States and the Philippines.

In a presentation at the July 2006 Third Annual Dragon Programme Symposium in Lijiang, China, van der A and colleagues including Jeroen Kuenen reported that NO_x_ emissions from China alone between 1997 and 2005 contributed to a small but important increase in ground-level ozone around the entire northern hemisphere, averaging roughly 0.3–0.5 ppb by volume when the air mass reached western North America, and about 0.2 ppb by volume when it drifted to Greenland, Europe, and northern Africa.

Several studies have found a strong correlation between satellite measurements and ground-level concentrations of NO_2_, says Randall Martin, an associate professor of physics and atmospheric science at Dalhousie University in Halifax, Nova Scotia. However, he says more study is needed to cross-check satellite data with ground monitors, develop better algorithms, and better address variables such as geographic location, seasonal effects, and atmospheric conditions. But Martin and colleague Lok Nath Lamsal say they’re getting close to more accurately determining ground-level concentrations via satellite, having developed algorithms that resulted in an 86% correlation between satellite and ground measurements of NO_2_ in favorable circumstances. Their report of these data has been accepted for publication in the *Journal of Geophysical Research*.

Because of its direct and indirect impacts on health and the environment, and spectral properties that make it relatively easy to track, CO has been monitored globally for a number of years. All or parts of all continents contain major emission generators at one time of year or another, and the transported CO adds to the load at certain times on all continents.

CO is created primarily by incomplete fuel combustion. Primary sources, especially in developed areas, include vehicles and various industrial processes. Natural and human-set vegetation fires also are a significant source. The concentrations of CO that are added to local settings via long-distance transport usually aren’t considered an added health burden on their own, Schoeberl says, but he notes that CO is “a great tracer for human activities and biomass burning.”

CO also plays a role in ground-level ozone formation. Several studies have found that transport of CO from one location is associated with increased ground-level ozone thousands of miles away. Examples include transport from North America to Europe, from Alaska and western Canada to Houston, Texas, and from Asia to western North America. Atmospheric CO also affects a number of other chemical reactions in the atmosphere.

Ground-level ozone is created primarily through reactions among NO_x_, VOCs, and other chemicals in the presence of sunlight. Ozone can cause health problems such as premature death and a range of respiratory and cardiovascular disorders.

Studies conducted over the past 15 years or so that are based on airplane data, ground monitors, and other instruments have provided substantial evidence that long-distance transport of ozone can affect other countries and continents. For instance, a report by Arlene M. Fiore and colleagues in the 15 August 2002 *Journal of Geophysical Research* noted that transport from outside North America can boost ground-level ozone by 15–35 ppb on summer afternoons in the United States. These imports of ozone can, in some places on some days, spike levels in some counties above the EPA standard of 75 ppb.

The long-distance spread of ozone has played a role in the large increase in ozone concentrations in many areas of the planet since about 1950, according to Roxanne Vingarzan, a senior scientist with Environment Canada. In a July 2004 *Atmospheric Environment* article she reported that ozone concentrations around the globe have roughly doubled since then. As scientists become more adept at using satellite imagery to track long-distance ozone movement within the troposphere and down to ground level, more detail should become available.

But the spectral properties of ozone make it difficult to take advantage of satellite instruments to track ground-level concentrations. “We have had satellites measuring total column ozone for some time,” Keating says. The problem, he explains, is that 90% of the ozone is in the stratosphere, so “measuring ozone in the troposphere requires looking through the stratosphere for the proverbial needle in a haystack.”

Schoeberl says the solutions to this problem likely lie in better algorithms to analyze the satellite data, as well as in new instruments aboard satellites. But he expects it may be a decade or so before there is a new ozone instrument in orbit.

For now, satellite images tracking ozone at an altitude of about 3 to 8 miles suggest that ozone is coming largely from developed countries in the northern hemisphere and from biomass burning (sometimes linked with human activities such as agriculture) in the southern hemisphere. The highest concentrations tend to occur in the summer months, although some areas—such as eastern China, California, and southwestern Africa—can have elevated concentrations in other months.

## A Glimpse of the Elusive VOCs and SO_2_

Vehicles, power plants, industrial processes, and consumer products are major human sources of VOCs, and vegetation is an important natural source. Most VOCs considered important from a health perspective—such as benzene, trichloroethylene, and chloroform—tend to have spectral properties that are hard to distinguish by satellite.

Formaldehyde is the only major VOC that’s readily detected via satellite, says Gunnar Schade, an assistant professor in atmospheric sciences at Texas A&M University in College Station. Its presence generally signals hydrocarbon sources such as forests, biomass burning, vehicles, and industrial processes that emit formaldehyde precursors such as ethene, isoprene, and methane. Deciduous forests tend to be the largest source of formaldehyde detected by satellites, though urban hotspots also show up.

Formaldehyde is short-lived in the atmosphere, but global images show modest long-distance transport at lower concentrations. The concentrations tracked by satellite “are not a real health concern,” Schoeberl says. However, formaldehyde and other VOCs do contribute to the formation of ground-level ozone. Although VOCs pose a variety of known human health risks, it’s hard to say whether the concentrations in the atmosphere that can be tracked by satellite pose those same risks.

SO_2_ is created primarily through combustion processes in power plants and various industrial processes such as pulp and paper mills. Important natural sources include volcanoes and biomass burning. Health effects include premature mortality, multiple respiratory problems, headache, nausea, and thyroid system disruptions.

Like VOCs, SO_2_ is difficult to track via satellite because of its spectral properties. Only major events such as volcanic eruptions and concentrated sources such as major urban areas tend to show up. It’s even harder translating atmospheric contamination into surface levels. “SO_2_ on the ground has been a challenge,” Haynes says, although recent advancements have allowed near-ground retrievals of SO_2_ from such major events.

Significant breakthroughs in satellite instruments or algorithms don’t appear to be on the near horizon. However, SO_2_ remains a concern for long-range transport. In an article by Chulkyu Lee and colleagues in the March 2008 issue of *Atmospheric Environment*, a team of Korean and German researchers using satellite data as one tool concluded that Chinese sources of SO_2_ boosted the pollutant at ground level at a Korean location in May 2005 by up to 7.8 ppb. That’s a significant portion of the EPA annual standard of 30 ppb or the 24-hour standard of 140 ppb.

## The Golden Age?

Satellite instruments may become increasingly important for tracking long-distance air pollutant transport, filling in data gaps between monitors, cross-checking ground-based emissions estimates and measurements, forecasting air quality, providing advance alerts for health care professionals, and supplying additional information for regulators as well as people and organizations trying to meet regulations. Along with information on air pollutants, satellite information is also being used to help with other important environmental health concerns, such as the spread of infectious diseases (as reflected by changes in vegetation and temperature, which can influence disease vector populations), dust, heat, land use changes, and climate change.

On the basis of what he’s seen so far, Keating says satellites just might pan out for the EPA in terms of monitoring air pollution. “I think there are some potential national applications. But it’s still in the developmental process.”

Part of that process includes plans by NASA, ESA, and others for about a dozen new satellites in various stages of conceptualization, planning, or design that would include air pollutant – tracking instruments. These could be launched anytime from 2009 to 2020 and beyond, and could replace and possibly improve upon the current fleet of at least nine satellites that track pollutants. Keating says the EPA is currently talking with NASA about specific pollutant tracking needs.

Nonetheless, competing interests and funding limitations may hinder the growth of the Earth-observation field, including pollutant-tracking efforts. On 14 January 2004 President Bush announced “a new vision for the Nation’s space exploration program,” emphasizing human and unmanned exploration of our solar system. Much of the initial focus is on returning to the moon and staying for extended periods, then going to Mars and eventually elsewhere. Little additional money has been budgeted for these projects so far, so the funds will come primarily out of existing NASA programs. “Earth science is a little on the back burner,” says Schoeberl. “NASA isn’t quite sure what it wants to do [given] the White House’s new focus on Mars.”

With launch dates for satellites routinely being pushed back years at a time, and with changing government priorities, Keating says the current fleet may be as good as it gets. “We’re already in the golden age of atmospheric chemistry information,” he says. “[But] we wonder how long these satellites will be in play. We’re very concerned there might be a dry period.”

## For More Time-Series Images

### NASA Earth Observatory

http://earthobservatory.nasa.gov/Observatory/datasets.html

Access data sets for selected pollutants back to 1978

### NASA Giovanni

http://disc.sci.gsfc.nasa.gov/techlab/giovanni/

Create customized views of remote sensing data

### ESA Tropospheric Emission Monitoring Internet Service

http://www.temis.nl

View near real-time data on pollutants and transport

### Global Monitoring for Environment and Security

http:/www.gse-promote.org

http://gems.ecmwf.int

Obtain global and European daily forecasts and daily and monthly archives for selected pollutants

### World Data Center for Remote Sensing of the Atmosphere

http://www.wdc.dlr.de

View near real-time stills and animations for pollutants

## Figures and Tables

**Figure:**
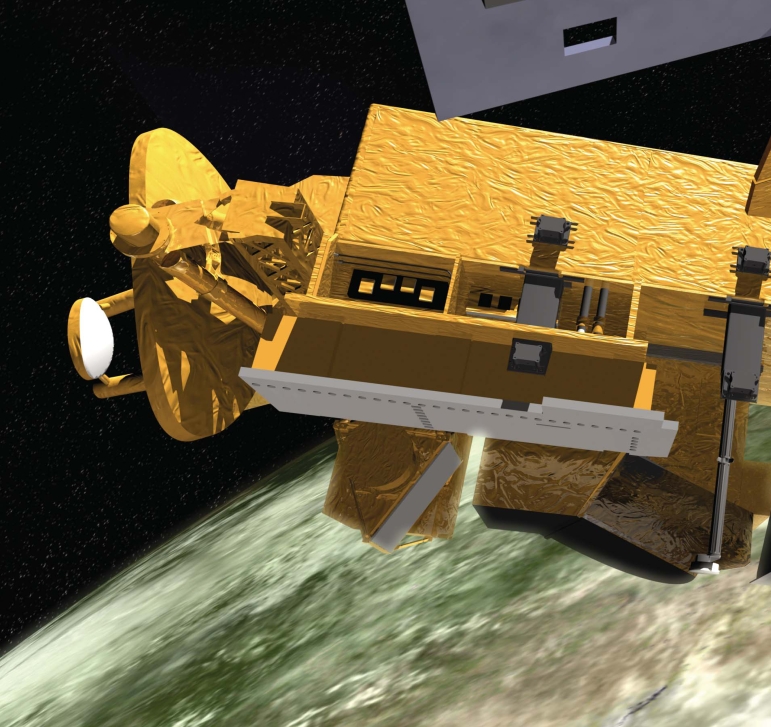
An artist’s rendering of the Aura satellite, which is dedicated to collecting data on air pollutants (image courtesy of NASA)

**Figure:**
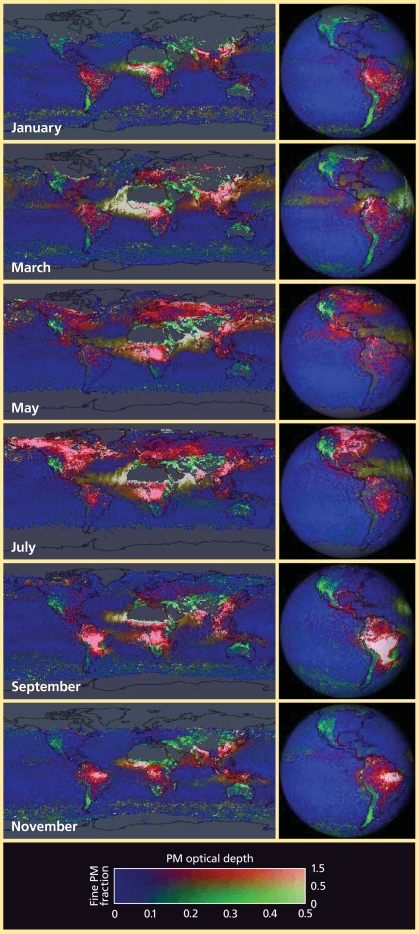
PM travels hundreds and thousands of miles, as seen in these satellite images for 2004 that illustrate PM density from the Earth’s surface to the top of the troposphere (which ranges from about 4 to 12 miles). Reds generally indicate human-generated sources, greens indicate primarily natural sources, and browns indicate a blend. Darker colors indicate greater concentrations. Atmospheric concentrations tend to be strongly to moderately correlated with ground-level concentrations, depending on local conditions. By carefully quantifying how much visible and near-infrared sunlight a PM plume reflects back up into space, scientists can accurately estimate the average size of the individual particles within the plume. Particles of natural origin (such as windblown dust) tend to be larger in size than human-produced particles, which mostly originate from the process of combustion (from fires or factories) and are therefore broken down into smaller particles. Source: NASA

**Figure:**
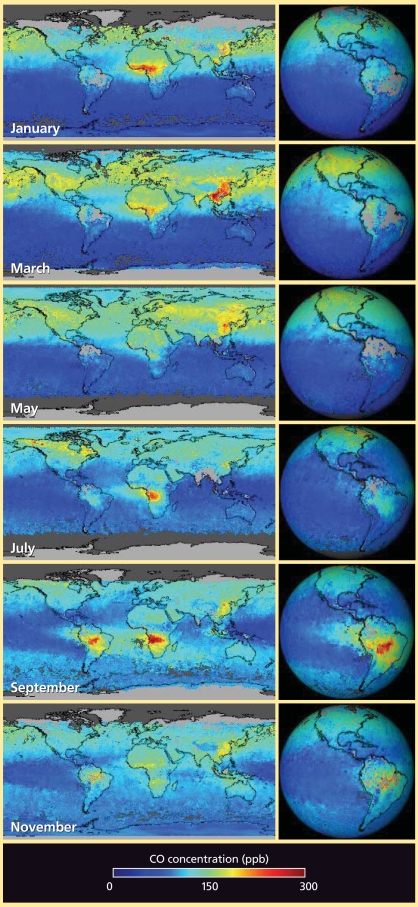
CO spreads hundreds and thousands of miles, as seen in these images of concentrations at about 12,000 feet. The data were collected in 2004 by the MOPITT (Measurements of Pollution in the Troposphere) sensor on NASA’s Terra satellite. Red indicates higher concentrations, and purple indicates lower concentrations. These concentrations tend not to pose a direct health threat, but serve as a tracer for a variety of other pollutants that can. CO, produced when carbon-based fuels burn incompletely or inefficiently, is one of the six major air pollutants regulated in the United States and in many other nations around the world. The amounts and sources of atmospheric CO change with locale and season. In Africa, for example, the seasonal shifts in CO are tied to the widespread agricultural burning that shifts north and south of the Equator with the seasons. Source: NASA

**Figure:**
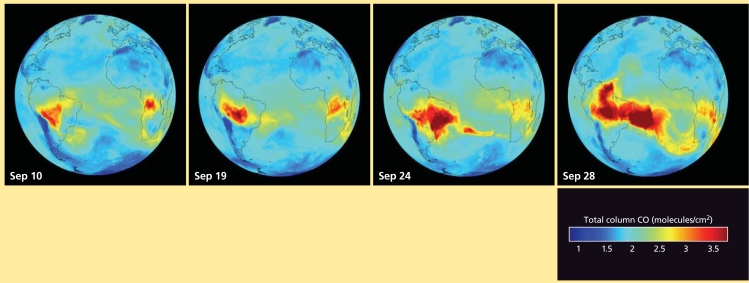
This image sequence taken over an 18-day period in September 2005 shows CO from agricultural fires blooming repeatedly over the Amazon basin, then traveling across the Atlantic Ocean, where it meets CO from fires in sub-Saharan Africa. These images came from the Atmospheric Infrared Sounder Experiment, whose visible, infrared, and microwave detectors provide a three-dimensional map of temperature, humidity, cloud cover, greenhouse gases, and other atmospheric phenomena. Source: NASA/JPL

**Figure:**
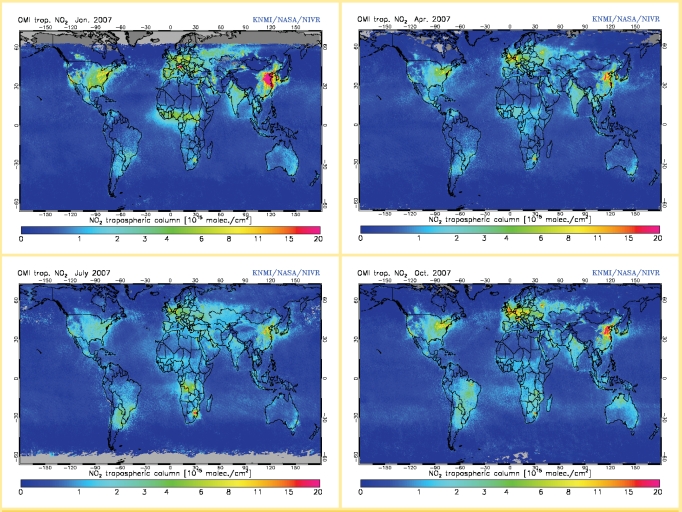
NO_2_ readily travels hundreds of miles at higher concentrations, and can travel thousands of miles at moderate or lower concentrations, as shown in this series of images from 2007. Red indicates the highest density, and purple the lowest, for the full column from the surface to the top of the troposphere. There can be a fairly strong correlation between atmospheric and ground-level concentrations, depending on local conditions. Source: ESA

**Figure:**
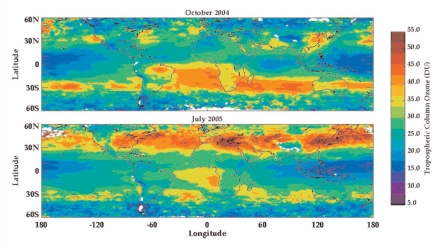
These images reflect relative ozone concentration in the middle troposphere, about 3 to 8 miles above the Earth’s surface. Red indicates higher concentrations, and purple indicates lower concentrations. It remains challenging to extend these data to layers closer than about 1 mile above the surface, but these images illustrate the major sources and general movement patterns of ozone, which blankets areas for up to thousands of miles. Short-term experimental global forecasts of surface-level ozone are available at http://gems.ecmwf.int/d/products/grg/realtime/daily_fields/. Source: NASA

